# Small bowel intussusception with the Meckel's diverticulum after blunt abdominal trauma: a case report

**DOI:** 10.1186/1749-7922-4-18

**Published:** 2009-05-06

**Authors:** El Bachir Benjelloun, Abdelmalek Ousadden, Karim Ibnmajdoub, Khalid Mazaz, Khalid Ait Taleb

**Affiliations:** 1 Department of general surgery, Universitet hospital Hassan II, Fes, Morocco

## Abstract

Intussusception with the Meckel's diverticulum is a rare but well-known cause of small bowel obstruction in the adult. After blunt abdominal trauma, intussusception is exceedingly rare and has been reported previously only in few cases. We present a case of a previously healthy 28-year-old man developing four days after blunt abdominal trauma signs of small bowel obstruction. Ileo-ileal intussusception was suggested by computed tomography. Exploration revealed ileo-ileal intussusception with Meckel's diverticulum. A diverticulectomy with small bowel resection was performed.

## Introduction

Intususception is invagination of a proximal segment of bowel (intussusceptum) into the lumen of the adjacent distal segment (intussuscipiens). While intusussception is relatively common in the childhood, it is infrequently seen in adults [1]. Whereas most cases in childhood occur idiopathically, in adults, an underlying cause is present in 80% of cases [2]. Causes include tumours and polyps as well oedema and fibrosis from recent or previous surgery, and Meckel's diverticula. Cases following blunt abdominal trauma are rare. We present a case of 28-year previously healthy man presenting with abdominal pain and vomiting after blunt abdominal trauma, and developing four days later signs of small bowel obstruction as a cause of ileoileal intussusception with the Meckel's diverticulum. From an extensive review of the literature, intussusception at the site of a Meckel's diverticulum following blunt abdominal trauma has not been previously reported.

## Case report

**Figure 1:**
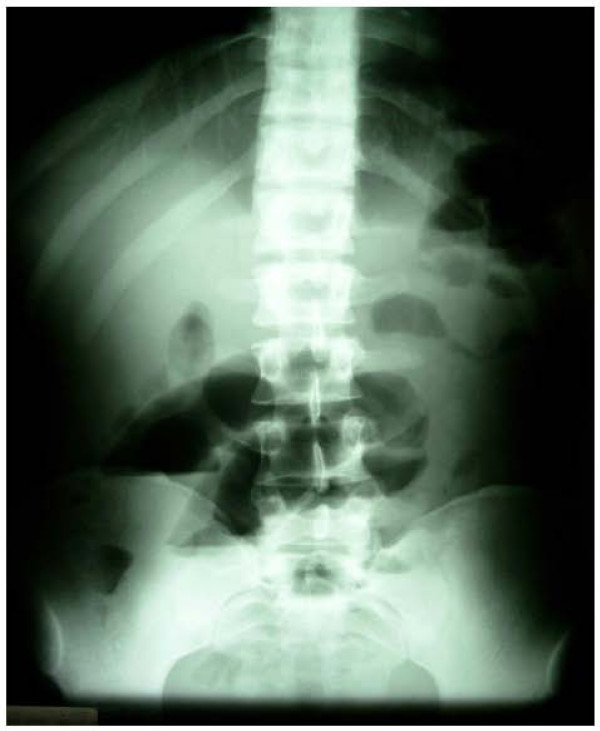
**X-ray shows multiple loops of dilated small bowel.with air- fluid levels**.

**Figure 2:**
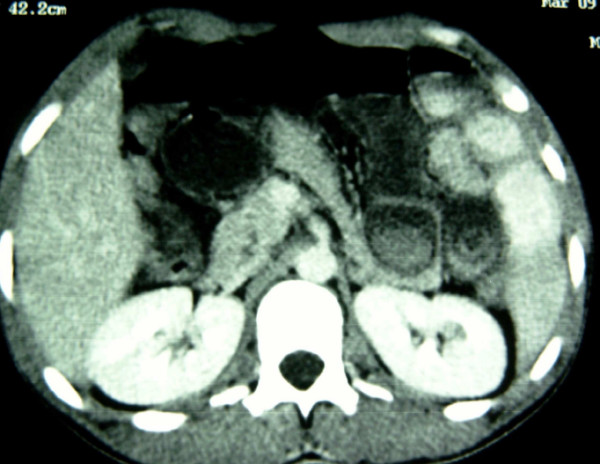
**CT of the abdomen demonstrating classic target sign in the the left upper quadrant, pathognomonic for ileoileal intussusception**.

**Figure 3:**
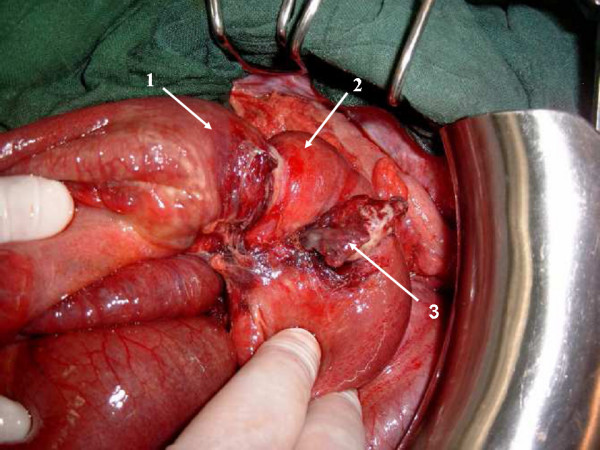
**Ileo-ileal intussusception with Meckel's diverticulum. 1- intussusceptum; 2- intussuscipiens; 3- Meckel's diverticulum**.

A 28-year-old previously healthy man presented at the emergency department (ED) 48 hours after a hit in the left side of the abdomen by a fist, with gradual worsening of pain, nausea and bilious vomiting. Physical examination revealed a temperature of 37,6°C, a pulse rate of 80 beat per minute (bpm), a blood pressure of 110/70 mm Hg. The epigastrium, left upper and left lower abdominal quadrants were tender on palpation. On rectal examination the rectum contained no stool. Initial management of the patient involved intravenous fluid resuscitation, and nasogastric tube insertion, routine blood tests and supine abdominal x-rays. Initial laboratory values, including complete blood cell count, serum electrolytes, glucose, blood urea, creatinine, liver function tests, and lipase were all normal. Initially supine abdominal x-ray revealed dilated small-bowel loops with air-fluid levels, but no gas under diaphragm (Fig. 1). Ultrasonography (US) of the abdomen showed free fluid in the peritoneal cavity with dilated small bowel loops without injuries of the parenchymatous abdominal organs. Diagnosis of hemoperitoneum was made, but with stability of vital signs, little abdominal tenderness, no signs of evident small bowel obstruction, and normal value of blood cell count, the patient was admitted in the surgery department for observation. During his hospital course his abdomen remained a little distended, with mild lower quadrant pain that was well controlled with analgesic pain medications. A repeat white and red blood cell count remained normal. Two days later, however, the abdominal pain was increasing, the vomits had turned fecaloid, and with absolute constipation. An abdominal computed tomography (CT) was performed which showed a targetlike lesion in the left upper quadrant with dilated small bowel loops proximally, suggestive of an ileo-ileal intussusception (Fig. 2). Free fluid was seen in the paracolic gutters, pelvis and between bowel loops. There was no solid organ injury. Based to the clinical signs of small bowel obstruction and the result of CT, an emergent laparotomy was decided. At the exploration, the peritoneal cavity was filled with 500 cc of blood-stained serous fluid, while numerous dilated loops of small bowel were present. At approximately 90 cm from the ileo-ceacal junction, there was an ileo-ileal intussusception with a small mesenteric breach likely accountable of blood stained. There was no solid organ injury. The intussusceptum was gently milked out, revealing a 20-cm segment of ischemic ileum. The intussuscepted segment was edematous, but there was no bowel wall hematoma. Just proximal to this, at approximately 100 cm from the ileo-ceacal junction, a small dimple in the antimesenteric border was noted and proved to be a Meckel's diverticulum (Fig. 3). Localized ileal resection with Meckel's diverticulum was undertaken. The postoperative recovery was uncomplicated, and the patient was discharged on the fifth day postoperatively. The pathological examination of the resected specimen showed a Meckel's diverticulum (3.5 cm in length) on the antimesenteric border with heterotrophic gastric mucosa.

## Discussion

Intussusception is defined as the telescoping of one segment of the gastrointestinal tract into an adjacent one. It is relatively common in children and is the second most common cause of an acute abdomen in this age group. It is much less common in adults and accounts for less than 5% of cases of mechanical small bowel obstruction [3].

However, blunt abdominal trauma is an unusual cause and only a few isolated cases reports are available in the literature. The underlying mechanism of traumatic intussusception is unknown but has been proposed to be a pathological peristaltic wave and/or localized spasm of a bowel segment after the trauma [4]. Another possible mechanism could be an intramural hematoma or edema acting as a lead point.

Meckel diverticulum is the commonest within a large number of lead points of structural, vascular/hematological, neoplastic, or inflammatory character [5]. In our case we think that the focal lead point was a Meckel's diverticulum, but a trauma with disturbed bowel motility was the unlatching mechanism of intussusception.

Adults will have a variable presentation of intussusception, often with a chronic colicky pain and intermittent partial intestinal obstruction associated with nausea and Vomiting [6], Because of this variable presentation, the diagnosis is often late.

An experienced hands ultrasound has both high sensitivity and specificity in the detection of intussusception. Classic findings on transverse scanning include a so-called "target lesion" or "doughnut sign", with the presence of several concentric rings [7]. CT is important in diagnosing associated pathology such as lymphadenopathy, it is often not very successful in determining the specific cause of the intussusception, as the lead point in many cases is small and often hidden within the intussuscepted mass [8].

All adult patients with intussusception will therefore require laparotomy. Resection is indicated in cases of large bowel intussusception, but reduction without resection may be an option in cases of small bowel involvement where the incidence of malignancy is not great and no abnormality of the small intestine is observed [9].

In conclusion, intussusception, although rare, should be considered when patients with blunt abdominal trauma present with insidious signs of obstruction.

## Consent

Written informed consent was obtained for publication of this case report and accompanying images. A copy of the written consent is available for review by the Editor-in-Chief of this journal.

## Competing interests

The authors declare that they have no competing interests.
